# The effectiveness of virtual reality in reducing preoperative anxiety among oral surgery patients under general anesthesia: a salivary stress biomarker and questionnaire-based study

**DOI:** 10.1186/s12903-026-08466-5

**Published:** 2026-05-06

**Authors:** Abel Tasman Yuza, Agus Nurwiadh, Indra Hadikhrisna, Arlette Suzy Setiawan

**Affiliations:** 1 Department of Oral and Maxillofacial Surgery, Faculty of Dentistry, Universitas Padjadjaran, Bandung, Indonesia; 2 Department of Pediatric Dentistry, Faculty of Dentistry, Universitas Padjadjaran, Bandung, Indonesia

**Keywords:** Virtual reality, Preoperative anxiety, Salivary cortisol, Alpha-amylase, APAIS

## Abstract

**Background:**

Preoperative anxiety is a common psychological response to surgical operations affecting the emotional, cognitive and physiological state of the patient. It can be measured by both objective and subjective methods. While the objective methods such as salivary alpha amylase and cortisol levels can give information about the physiological state of the patient, the subjective assessment of anxiety can be measured by questionnaires such as Amsterdam Preoperative Anxiety and Information Scale (APAIS). However, the methods used to treat preoperative anxiety are evolving.

**Objective:**

This study aimed to investigate the effect of Virtual Reality (VR) on preoperative anxiety in patients undergoing the extraction of all four third molars.

**Methods:**

A comparative study involving 30 patients who need extraction of all four third molars, randomly assigned to a control or VR group (15 participants per group), was conducted. The VR group received a 15 min immersive VR relaxation session prior to anesthesia induction, while the control group underwent standard preoperative waiting for the same duration. Salivary alpha-amylase and cortisol levels were measured pre- and postintervention measured using enzyme-linked immunosorbent assay (ELISA), and anxiety was assessed via the APAIS. Statistical analysis included paired and independent t-tests.

**Results:**

Post-intervention APAIS scores were significantly lower in the VR group than in the control group (*p* < 0.001). At the end of the experiment, the VR group had lower levels of salivary amylase compared to the control group. However, there was no significant difference between the groups with respect to changes in salivary amylase from pre-intervention (baseline) to post-intervention (after 3 weeks). There was also a decrease in salivary cortisol levels from pre-intervention to post-intervention in both groups. However, there was no significant difference in salivary cortisol levels between the two groups. In summary, VR is a promising, safe, and non-drug method for reducing pre-operative anxiety, demonstrated by significant reductions in salivary amylase levels and APAIS scores. VR has also helped improve patients’ comfort and psychological readiness for undergoing oral surgery, although no significant difference between salivary cortisol levels was found.

## Background

Anxiety is a state of discomfort characterized by feelings of worry, fear, and tension, often accompanied by behavioural, emotional, cognitive, and physiological responses [1, 2]. The preoperative period is a well-established trigger for anxiety, especially in patients undergoing oral surgery. Among these surgeries, the extraction of four impacted wisdom teeth is particularly likely to cause anxiety because of the significant surgical trauma, potential postoperative discomfort, and the unfamiliar surgical environment. This psychological stress can activate the sympathetic nervous system and the hypothalamic-pituitary-adrenal (HPA) axis, leading to elevated levels of salivary alpha-amylase and cortisol [3–5].

Research has been conducted on the use of VR-based therapies in different areas of medicine (i.2., plastic surgery, maxillofacial surgery, outpatient sedation) yet investigations into the use of VR for reducing anxiety levels amongst patients who have multiple third molars while under general anesthesia is lacking [6–8]. Several study have investigated patients’ level of anxiety related to receiving local anaesthesia and/ or VR as a method to distract patients from the pain while actively extracting the patient’s tooth, but have failed to research preoperative levels of anxiety from patients who are scheduled to undergo GA.

Furthermore, few studies have employed a multidimensional approach integrating physiological biomarkers and subjective assessments to evaluate preoperative anxiety. Salivary alpha-amylase and cortisol are recognised non-invasive acute stress indicators, reflecting activation of the autonomic nervous system and the hypothalamus-pituitary-adrenal (HPA) axis, respectively [9–11]. Combining these biomarkers with validated psychological assessment tool, such as the Amsterdam Preoperative Anxiety and Information Scale (APAIS), which assesses patients’ emotional and informational needs, provides a comprehensive framework for anxiety assessment [12, 13]. The APAIS is concise, validated for preoperative assessment, and more suitable for routine surgical practice compared to longer scales such as the State-Trait Anxiety Inventory (STAI) [14–16].

Therefore, the present study aimed to investigate the effectiveness of VR as a nonpharmacological, immersive intervention in reducing preoperative anxiety in patients undergoing full-mouth third molar extraction under general anaesthesia. By employing both salivary biomarkers and APAIS scores, this study seeks to expand the evidence base and highlight the potential of VR for anxiety management in oral surgical care.

## Methodology

The Research Ethics Committee at Universitas Padjadjaran granted this study ethical approval (965/UN6.KEP/EC/2024) prior to enrolling participants, and all research procedures were completed in compliance with the Declaration of Helsinki. This research study took place from September through December (3 months) in 2024.

### Sample size and sampling

The sample size was predetermined based on the expected effect size of d = 0.8, α = 0.05, and statistical power (1 − β) = 90%, ultimately requiring 15 participants per group.

### Study subjects

All participants were scheduled for full-mouth extraction of all four impacted third molars under general anaesthesia, a procedure categorized under minor oral surgery in our center due to its surgical complexity and psychological impact. The selection was based on explicit inclusion and exclusion criteria to ensure the reliability and safety of the intervention. Inclusion criteria were that patients had to be scheduled for extraction of all four third molars, be free of systemic disease, have no history of orthodontic surgery, not have participated in similar studies, and not have been exposed to virtual reality (VR).

Individuals with known endocrine disorders, current infections, or medications affecting salivary secretion or stress response were excluded to minimize potential confounding effects on cortisol and alpha-amylase levels. Exclusion criteria were patients with a history of psychiatric or neurological disorders, patients currently taking antianxiety medications or corticosteroids, patients with impaired salivary gland function, participants with oral or physical injuries at the time of assessment, and patients who did not or could not comply with the study procedures. Patients who may have had their virtual reality experience confounded due to vision and/or hearing impairments were also excluded from the study. The rationale behind the exclusion of these two criteria was to eliminate potential variability and create a VR intervention that would help to reduce surgical anxiety for patients undergoing surgery.

**Fig. 1:**
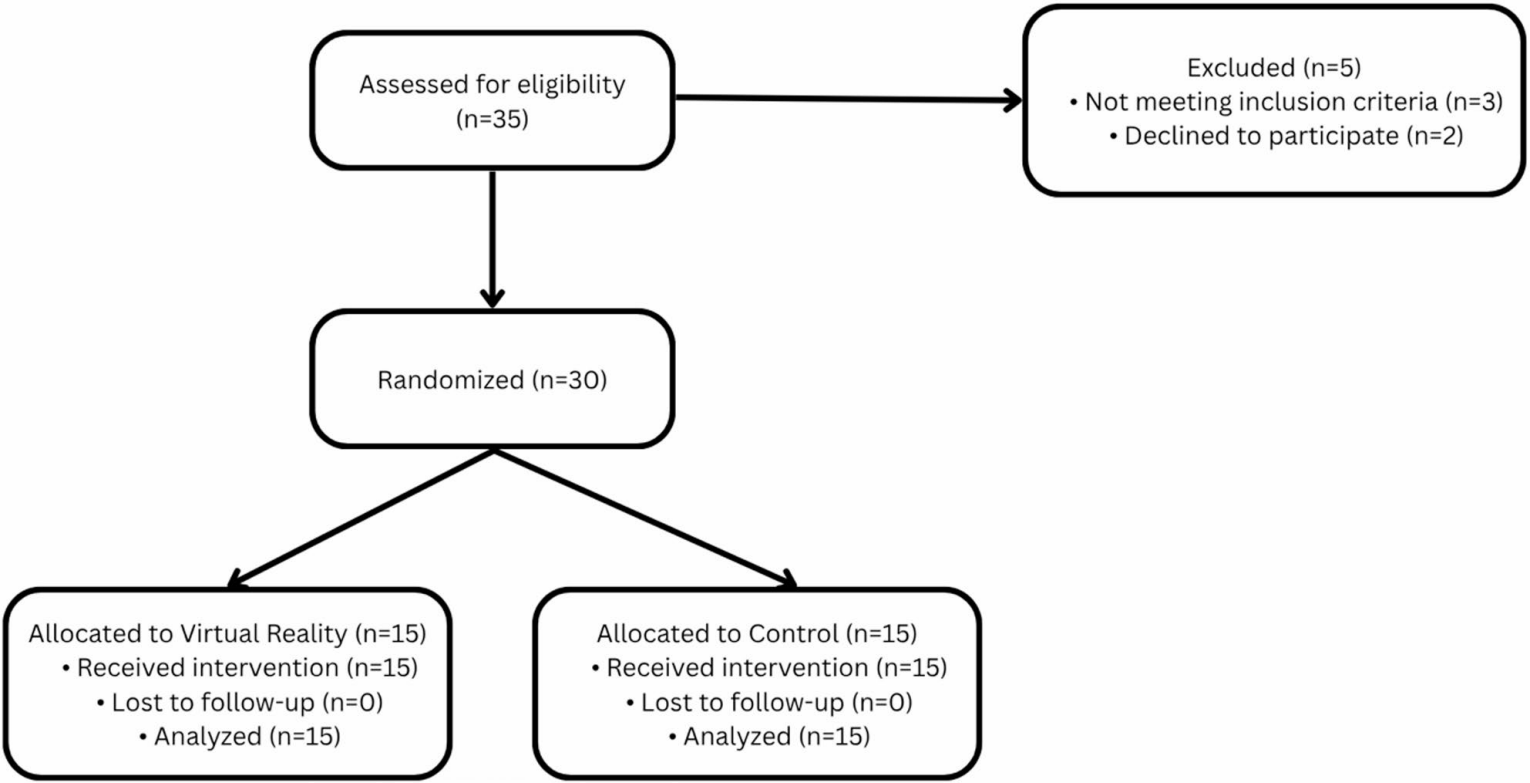
CONSORT Flow Diagram. Study participants go through all phases of the randomized controlled trial, including recruitment, allocation, follow-up, and analysis

Out of an initial 35 patient eligibility assessments, five patients were found to be ineligible and excluded from the study because they did not meet the inclusion criteria (*n* = 3) or declined to participate (*n* = 2). Each of the 30 remaining patients was randomly allocated into either the control group (*n* = 15) or the virtual reality group (*n* = 15). All participants completed the intervention and were included in the final analysis. The participant flowchart is shown in the CONSORT flowchart (Fig. 1).

In addition, participants could not have any oral or physical injuries and were required to provide written informed consent before participation. Participants were randomly assigned to either the virtual reality group or the control group. A sealed envelope method was used to conceal the assignment results and minimize selection bias. No stratification by age, sex, or other variables was applied during randomization; however, post-randomization analysis confirmed that the groups were comparable in baseline characteristics.

Participants assigned to the control group followed the standard preoperative protocol implemented at our institution. Prior to having anesthesia induced, they waited in a quiet waiting area for about 15 min. The environment was lit by ambient room light, furnished with typical clinical furniture, and did not have any multimedia devices to provide guided relaxation or social interactions beyond performing routine preoperative monitoring. No Audio or Video were played, nor was any Audio or Video provided with an agenda toward relaxation. There were no relaxation instructions given or practiced in the waiting room. Clinical staff provided only minimal verbal directions about the treatment procedure and typical routine general reassurances before beginning why there would be clinical staff with more than one physician at a time per routine protocols. This was intended to stimulate actual hospital wait times and serve as a benchmark for evaluating the effectiveness of the intervention group’s immersive VR experience.

Randomisation was conducted using a computer-generated sequence generated by the first author, who served as the principal investigator. To maintain allocation concealment, the randomization sequence was implemented using opaque, sealed, and sequentially numbered envelopes. Each envelope contained a task for one participant, which was prepared in advance by a research assistant who was neither involved in recruitment nor in outcome evaluation.

After obtaining written informed consent and completing baseline data collection, the research assistant—blinded to the randomization sequence—opened the next envelope in numerical order to determine the participant’s group assignment. This process was conducted immediately before the intervention began. The procedures were designed to minimise selection bias and ensure proper concealment of allocation throughout participant enrolment.

The APAIS questionnaire was administered twice during the preoperative period: (1) at baseline after group assignment and before any premedication, and (2) immediately after the 15 min VR or control condition, still before premedication and before transfer to the operating room. Premedication (benzodiazepines) was administered after completion of the second APAIS assessment and the second saliva collection. The APAIS contains six items scored on a 5-point Likert scale, yielding separate anxiety and information subscale scores. This questionnaire has gone through a transadaptation process into Indonesian with the Cronbach alpha reliability test showing high reliability values ​​(0.825 and 0.863) [17].

### Blinding

Due to the nature of the intervention, blinding of participants was not feasible. However, steps were taken to minimise bias in outcome assessment. Laboratory personnel performing Enzyme-linked immunosorbent assay (ELISA) analysis of salivary biomarkers (alpha-amylase and cortisol) were fully blinded to group assignment. In addition, data entry and statistical analyses were conducted by a separate team of researchers who were not involved in participant recruitment or group assignment. This partial blinding method is used to reduce bias in the detection and analysis of physiological and questionnaire results.

## Intervention procedures (VR Group and control Group)

### VR intervention

Participants in the VR group experienced a soothing VR environment approximately 15 min before surgery. The VR experience was passive (non-interactive), consisting of a standardized nature scene (tropical beach) with natural ambient sound, viewed through an Oculus Go headset with integrated stereo headphones. This combination of audiovisual stimulation was designed to promote relaxation and reduce anxiety associated with anesthesia induction.

### Control condition

Participants assigned to control group completed the regular waiting process before surgery without being exposed to the VR intervention. They spent about 15 minuts in the surgery preparation room, participated in standard pre-surgical monitoring, and received only routine information and reassurance pertaining to their surgery without any additional audiovisual orguided relaxation techniques.

### Saliva collection and ELISA analysis

To minimize the impact of circadian rhythms, two saliva samples were collected between 8:00 AM and 10:00 AM. The first (baseline) sample was collected in the preoperative holding area before exposure to the VR or control condition. The second (post-intervention) sample was collected 1immediately after completion of the VR or control period and before anesthesia induction. Unstimulated saliva samples was collected using the passive drool technique over 5 min. Saliva samples were collected twice: baseline sample – collected in the preoperative holding area before exposure to VR or control condition, and preoperative sample – collected after 15-minute intervention period, just before transfer to the operating room. Samples (4–5 mL) was collected and stored at 4 °C (for up to 24 h) or − 10 °C for later analysis. ELISA (Salimetrics, USA) procedures followed standard protocols, including the use of a cortisol‒HRP conjugate, detection reagents, a substrate solution, and a microplate reading at 450 nm.

### Data analysis

The data were coded, tabulated, and analyzed via SPSS v25.0 (IBM Corp., Armonk, NY, USA). Normality was assessed using the Shapiro–Wilk test. Given the repeated-measures randomized design (two groups measured at two time points), the primary treatment effect was evaluated using a mixed-design (two way) repeated-measures analysis testing the Group x Time interaction for each outcome (APAIS, salivary alpha-amylase, and salivary cortisol), with surgical duration entered as a covariate where applicable. Where model assumptions were not met, a linear mixed-effect model with fixed effect for group, time, and their interaction (and a random intercept for participant) was considered. Paired t-tests (within-group) and independent t-tests (between-group) were reported as supportive descriptive analyses. Pearson’s correlation coefficients were calculated to explore association between APAIS scores and salivary biomarkers. All tests were two-sided, with *p* < 0.05 considered statistically significant. Effect sizes were reported as Cohen’s d for between-group comparisons (small = 0.2, medium = 0.5, large = 0.8). Given the pilot sample size, effect size estimates may be inflated and should be interpreted cautiously.

## Results

### Participant characteristics and surgical overview

All 30 patients (15 in the VR group and 15 in the control group) undergoing full-mouth extraction of all four impacted third molars under general anesthesia completed the study without any intraoperative or postoperative complications. The duration of surgery ranged from 20 to 100 min, with the majority lasting 30 to 45 min (mean 38.0 *±* 18.4 in control group; 42.3 *±* 21.7 in VR group). Despite these variations in operative time, the absence of complications across all cases indicates that the procedures were conducted safely and efficiently.

Participants were aged 18 to 30 years. Most were university students or young professionals from urban backgrounds. Randomisation was used to ensure baseline comparability, and no significant differences were observed between the VR and control groups in age, sex, or educational background (Table 1).

**Table 1:** Baseline characteristics of participants in the control and VR groups

Characteristic	Control Group (*n* = 15)	VR Group (*n* = 15)	*p*-value
Age (years)	23.2 ± 3.3	24.3 ± 3.7	0.383¹
Sex [n (%)]			0.699²
• Male	6 (40.0%)	4 (26.7%)	
• Female	9 (60.0%)	11 (73.3%)	
Occupation/Status [n (%)]			0.812²
• University student	12 (80.0%)	11 (73.3%)	
• Young professional	3 (20.0%)	4 (26.7%)	
Surgical duration (Mean ± SD)	38.0 ± 18.4 min	42.3 ± 21.7 min	*p* = 0.560

¹Independent t-test, ²Chi-square test

All primary outcomes are reported with p-values, 95% confidence intervals, and effect sizes (Cohen’s d) to enhance interpretability and clinical relevance.

For salivary alpha-amylase (U/mL), post-intervention levels were lower in the VR group (37.0 ± 9.1 U/mL) than in the control group (77.4 ± 12.0 U/mL); however, the mixed-design repeated-measures model did not demonstrate a significant Time x Group interaction (*p* = 0.208), indicating that the magnitude of pre–post change did not differ significantly between groups. Thus, alpha-amylase findings should be interpreted as an overall preoperative reduction rather than a confirmed VR-specific physiological effect. Detailed values are presented in Table 2 and the interaction results are summarized in Table 6.

**Table 2:** Differences in the levels of alpha-amylase between the control and VR groups

Variables	Control (Mean ± SD)	Virtual Reality (Mean ± SD)	Mean Difference (95% CI)	*p*-value	Cohen’s d	Interpretation
Pre-intervention	177.6 ± 49.8	156.6 ± 43.7	21.0 (− 13.6 to 55.6)	0.228	0.45	Small effect
Post-intervention	77.4 ± 12.0	37.0 ± 9.1	40.4 (32.8 to 48.0)	< 0.001	3.79	Very large effect
Difference score	100.2 ± 43.7	119.5 ± 44.8	−19.3 (− 52.4 to 13.8)	0.241	0.44	Small effect

### Bottom of form

Salivary cortisol decreased from pre- to post-intervention in both groups’. Post-intervention cortisol was lower in the VR group than in the control group (*p* < 0.001); however, the between-group difference in change scores (post-pre) was not statistically significant (*p* = 0.451). The time x Group interaction in the repeated-measures model was not significant (*p* = 0.426). Therefore, these findings do not support a VR-specific physiological effect on cortisol beyond routine care in this pilot sample. Refer to Table 3 for detailed statistics and Table 6 for interaction model.

**Table 3:** Differences in salivary cortisol levels between the Control and VR groups (ng/mL)

Variables	Control (*n* = 15) Mean ± SD	VR (*n* = 15) Mean ± SD	Mean diff (Control-VR)	95% CI	*p*-value
Pretest	16.1 ± 9.7	15.6 ± 9.2	0.46	-6.62739 to 7.54072	0.896
Posttest	4.2 ± 1.0	1.3 ± 1.0	2.91	2.18533 to 3.62801	< 0.001
Change (Post-Pre)	-11.8 *±* 9.1	-14.3 *±* 8.4	2.45	-4.11169 to 9.01169	0.451

Independent-samples t-test (equal variance assumed; Levene’s test *p* > 0.05 for all rows)

APAIS scores were comparable at baseline (*p* = 0.883), but post-intervention scores were significantly lower in the VR group than in controls (*p* < 0.001), and the change score (post-pre) differed significantly between groups (*p* = 0.007), consistent with the significant Time x Group interaction observed in the repeated-measures model. Refer to Table 4 for detailed statistics (and Table 6 for the interaction model).

**Table 4:** Differences in APAIS scores between the control and VR groups

Variables	Control (*n* = 15) Mean ± SD	VR (*n* = 15) Mean ± SD	Mean diff (Control-VR)	95% CI	*p*-value
Pretest	19.5 ± 2.5	19.3 ± 2.4	0.13	-1.70462 to 1.97129	0.883
Posttest	17.8 ± 1.2	14.7 ± 2.0	3.13	1.91956 to 4.34711	< 0.001
Change (Post-Pre)	-1.67 ± 3.04	-4.67 ± 2.53	3.00	0.90975 to 5.09025	0.007

Independent-samples t-test (equal variance assumed; Levene’s test *p* > 0.05 for all rows)

Table 5 summarizes the within-group changes in anxiety-related outcomes before and after the intervention, including APAIS scores and salivary biomarkers, to illustrate the magnitude and statistical significance of pre–post differences within each study group.

**Table 5:** Within-Group Comparison of Anxiety Scores and Biomarkers (Pre–Post Intervention)

Group	Outcome	Pre mean *±* SD	Post mean *±* SD	Δ (Post-Pre), mean *±* SD	*p*-value
Control	APAIS score	19.5 *±* 2.5	17.8 *±* 1.2	-1.7 *±* 3.0	0.052
VR	APAIS score	19.3 *±* 2.4	14.7 *±* 2.0	-4.7 *±* 2.5	**< 0.001**
Control	Alpha-amylase (U/mL)	177.6 *±* 49.8	77.4 *±* 12.0	-100.2 *±* 43.7	**< 0.001**
VR	Alpha-amylase (U/mL)	156.6 *±* 43.7	37.0 *±* 9.1	-119.5 *±* 44.8	**< 0.001**
Control	Cortisol (ng/mL)	16.1 *±* 9.7	4.2 *±* 1.0	-11.8 *±* 9.1	**< 0.001**
VR	Cortisol (ng/mL)	15.6 *±* 9.2	1.3 *±* 1.0	-14.3 *±* 8.4	**< 0.001**

Values are mean ± standard deviation

*p*-values based on paired t-tests comparing pre- and post-intervention within each group

Bolded *p*-values indicate statistically significant differences (*p* < 0.05)

*APAIS* Amsterdam Preoperative Anxiety and Information Scale

### Correlation analysis

At baseline, APAIS scores showed a moderate positive correlation with salivary cortisol (*r* = 0.39, *p* = 0.035), while the correlation with alpha-amylase was weaker and not statistically significant (*r* = 0.27, *p* = 0.147). After the intervention, APAIS scores were strongly correlated with both cortisol (*r* = 0.65, *p* < 0.001) and alpha-amylase (*r* = 0.66, *p* < 0.001). In contrast, changes in APAIS (ΔAPAIS) were not significantly correlated with changes in cortisol (*r* = 0.31, *p* = 0.096) or alpha-amylase (*r* = 0.20, *p* = 0.286). These correlations describe association at the measured time points and do not imply causality.

### Mixed-design repeated-measures analysis

Table 6 shows the mixed-design repeated-measures analysis (Time: pre vs. post; Group: VR vs. control), with surgical duration entered as a covariate, performed to evaluate the primary treatment effect (Time x Group interaction). For APAIS, there was a significant main effect of time (F(1,27) = 11.574, *p* = 0.002, partial eta squared = 0.300) and a significant Time × Group interaction (F(1,27) = 8.945, *p* = 0.006, partial eta squared = 0.249), indicating that the reduction in self-reported preoperative anxiety over time was significantly greater in the VR group than in the control group. The Time × Duration interaction was not significant (F(1,27) = 0.633, *p* = 0.433).

**Table 6:** Mixed-design repeated-measures analysis (Time × Group), adjusted for surgical duration

Outcome	Effect	F (df1, df2)	*p*-value	Partial eta squared
Cortisol	Time	F(1,27) = 15.834	*p* < 0.001	0.370
	Time × Group	F(1,27) = 0.653	0.426	0.024
	Time × Duration	F(1,27) = 0.274	0.605	0.010
Alpha-amylase	Time	F(1,27) = 45.667	*p* < 0.001	0.628
	Time × Group	F(1,27) = 1.666	0.208	0.058
	Time × Duration	F(1,27) = 0.880	0.357	0.032
APAIS	Time	F(1,27) = 11.574	0.002	0.300
	Time × Group	F(1,27) = 8.945	0.006	0.249
	Time × Duration	F(1,27) = 0.633	0.433	0.023

Time = pre vs. post. Group = VR vs. control. Surgical duration was included as a covariate. For two-level Time, sphericity corrections are equivalent

For salivary alpha-amylase (U/mL), the mixed-design repeated-measures model showed a strong main effect of Time (F(1,27) = 45.667, *p* < 0.001, partial eta squared = 0.628), indicating an overall decrease from pre to post across the entire sample. The interaction between time and group was not statistically significant (F(1,27) = 1.666, *p* = 0.208, partial η² = 0.058), indicating no significant difference in the degree of change of α-amylase over time between the VR group and the control group. The interaction between time and duration was also not significant (F(1,27) = 0.880, *p* = 0.357).

Likewise, there was a significant main effect (F(1,27) = 15.834, *p* < 0.001, partial η² = 0.37) for time on salivary cortisol levels (ng/ml), indicating a general decline in salivary cortisol levels over time in response to the intervention. In contrast, there was not a significant interaction effect for time and group (F(1,27) = 0.653, *p* = 0.426, partial η² = 0.024), indicating no differential effect of VR on changes in cortisol levels over time. The interaction of time and duration was not significant either (F(1,27) = 0.274, *p* = 0.605).

## Discussion

This controlled clinical study shows that virtual reality (VR) may provide an effective, safe, nonmedicinal method to mitigate preoperative anxiety. Individuals assigned to the VR condition displayed statistically significant decreases in both salivary alpha amylase (α-AmyLv) and the Amsterdam preoperative anxiety survey (APAIS) scores. Changes in cortisol levels were numerically lower for participants in the VR condition, indicating that individuals who used VR to reduce their anxiety experienced both physical and psychological changes in stress response. Further, participants assigned to the VR condition demonstrated statistically greater differences in APAIS scores before and after the intervention than did those assigned to the control condition. There were no statistically significant differences in the degree of change in salivary α-amylase or cortisol between groups.

The results support a broadening area of research demonstrating the impact of virtual reality (VR) upon the autonomic nervous system (ANS) and the hypothalamic-pituitary-adrenal (HPA) axis. Many previous studies have produced similar results demonstrating that immersive distracting environments can help decrease anxiety when used in multiple medical fields such as dentistry, oncology, and general surgical procedures [7–9].

### Physiological mechanisms and biomarker validity

Salivary alpha-amylase and cortisol are reliable, noninvasive biomarkers representing different stress pathways: alpha-amylase reflects rapid sympathetic activation, while cortisol represent HPA axis output, which responds more slowly to stress. The VR group showed lower values for all indicators after testing, especially a decrease in alpha-amylase, highlighting the added value of VR beyond routine preoperative care. Alpha-amylase responds particularly rapidly to psychological stress, as reported by AlMaummar et al. [6] and Jafari et al. [7] serving as a sensitive indicator of real-time anxiety regulation. This has been confirmed by studies by Vacaru et al. [18] and Chaturvedi et al. [11] Although cortisol levels decreased in both groups, the difference between groups was not statistically significant. This may be due to the delayed response of the HPA-axis to cortisol and its high bio variability.

After the intervention phase, alpha-amylase and cortisol levels in saliva were marked reduced for both groups; these results imply a reduction in levels of anxiety may be due to non-specific factors like: natural acclimatization to the pre-operative environment, placebo effects or general preparation of the psyche for anesthesia prior to being exposed to surgery. Procedures associated with preoperative preparation such as; given adequate amount of peaceful (quiet/not disturbed) waiting time prior to the operation time until they have their surgery performed) and providing for same generalized manner (standard Interview) with the clinical staff could provide for a reassurance that would aid to reduce in levels of stress biomarker(s). These factors should be considered when interpreting the added value of VR, as they underscore the importance of contextual and environmental influences in modulating preoperative anxiety. Dental anxiety also may played important role in the effects of the result on salivary biomarkers [19].

Although cortisol levels decreased from pre- to post-intervention in both groups, the between-group difference was not statistically significant. Therefore, cortisol findings should be interpreted cautiously in this pilot sample, particularly given cortisol’s delayed HPA-axis kinetics and inter-individual variability. The physiological secretion of cortisol follows a daily cycle and the possible impact from future stressors can produce variability between time and intensity for each person. Cortisol secretion is impacted by the collection timing of saliva for testing in relation to the use of virtual reality (VR) intervention as well as the start of the general anesthesia cycle as the specific time points for each event may not match the high or low secretion times of cortisol; therefore, any difference in cortisol from the degree of VR will be diluted. The small sample size may create a statistical power limitation and create difficulty in discovering any subtle differences in cortisol secretion and/or VR efficacy between groups. Although these caveats exist, the trend of decreased cortisol levels of the VR group post-testing indicates that further application within a larger multicenter study be warranted and pursued. An interesting finding is that cortisol levels in the VR group were significantly lower than before the application of testing, which agrees with the results of Pasyar et al. [8], as they reported similar results with aromatherapy and Lewandowski et al. [20], as they reported significant decreases in cortisol levels with infusion in those patients receiving VR therapy.

An important methodological consideration is the timing of cortisol measurement. Salivary cortisol reflects the activity of the HPA-axis response to acute psychological stress is typically delayed [21]. In this study, saliva samples were collected immediately after the intervention, i.e. immediately after the 15-minute VR/control phase and before anesthesia induction. This time point may not have captured the maximum cortisol response or the full curve of HPA-axis regulation, which limits the interpretability of the results and may have resulted in insignificant intergroup-time interactions of cortisol. Future studies should incorporate additional sampling points (e.g., 20–30 min post-intervention and/or early postoperative measures) to better characterize cortisol dynamics and detect potential VR-related physiological effects.

These findings suggest that, at a single time point, higher self-reported anxiety is consistently associated with higher salivary stress biomarker levels, particularly post-intervention. However, the lack of a significant correlation between changes in APAIS and changes in biomarkers suggests that, in this small pilot sample, short-term fluctuations in self-reported anxiety may not be linearly reflected in the dynamic changes in biomarkers.

### Subjective anxiety reduction

The APAIS scores reflect the subjective perception of anxiety and confirm that patients who received VR interventions felt significantly less anxious before surgery. Although the physiological measurement data were protected by blinding, the subjective decrease in APAIS scores may be partly attributed to the anticipation effect. This finding is consistent with biomarker results and suggests that VR immersive experiences have significant psychophysiological effects. Szczepańska-Gieracha et al. [22] and Meshkat et al. [23] reported that VR can reduce anxiety, stress, and discomfort in surgical patients and patients with chronic dieases. Our findings further expand on this understanding, confirming similar benefits of VR in oral and maxillofacial surgery—an area where anxiety often leads to treatment delays or increased pain.

VR has many benefits in clinical practice including ease of use, cost-effectiveness, no harm to patients, and ease of repetition. These features make it an optimal choice for assisting with patient-centered care by providing necessary psychological readiness, particularly where healthcare resources are limited (outpatient setting). Moreover, incorporating VR technology with standardized procedures could improve the quality of care delivered by enhancing patient experiences, adherence and ultimately result in improved surgery outcomes. These findings highlight the potential of VR as a practical, low-risk tool for managing preoperative anxiety in minor oral surgeries [23]. Because this study used an open-label design, the reduction in APAIS scores may be influenced by the expected or novelty effect.

In this randomized pre- and post-test design, the primary therapeutic effect was observed in the group-time interaction. A repeated measures analysis demonstrated a significant interaction of VR with APAIS scores indicating that VR was found to be superior to conventional preoperative care in decreasing preoperative anxiety. However, no group-and-time interaction occurred for salivary cortisol and α-amylase results; findings suggest there was no difference in the response of group salivary cortisol and α-amylase over time. It appears from these preliminary findings that the primary effect of VR during preoperative care was to assist patients in their ability to effectively manage their preoperative anxiety; however, the physiological responses of patients’ salivary stress biomarkers using VR were still uncertain at the present time. This discrepancy may be related to the high biological variability of salivary biosurfactant(s), the kinetics and circadian rhythm sensitivity of cortisol, as well as the influence of nonspecific situational factors (such as familiarity with prior surgery and routine reassurance) on both groups resulting in decreased physiological stress responses. In order to determine whether there are additional physiological benefits to VR, future studies must be larger, more reliable, utilize more frequent measurements and have better controls.

### Limitations

When interpreting the results of this study, remember that multiple limitations exist. First, sample size was small; additionally, participants were recruited from one center only. Although it is possible to use the sample size to perform an exploratory pilot study, relying only on this sample size may limit some ability to identify smaller or differentially affected outcomes in the sample. For instance, it is known that levels of cortisol (= stress) show a lot of variability between people and respond slowly (in relation to time) to stress, therefore, minus statistically significant differences, cortisol results from the present study should be interpreted carefully. Larger samples and more sites are needed to enhance generalizability and statistical power in future investigations.

Second, due to the nature of the intervention, an open-label study design was necessary because participants could not be blinded regarding their virtual reality (VR) exposure. This could introduce anticipation or novelty effects, particularly regarding the self-reported anxiety score (APAIS). To minimize bias, all participants were placed in a quiet, dimly lit environment before the procedure, and their saliva biomarkers were analyzed by laboratory personnel unaware of their group assignments. However, the possibility of anticipation bias cannot be completely ruled out.

Third, although randomizations have been completed for subjects, the study did not implement stratifications. Those subjects’ potential confounding variables like sex, age, psychological resilience, and history of surgical experience could also impact anxiety levels in both physiological and subjective ways among the different participant cohorts included in this analysis. Future research should look into using either stratified or multivariate approaches to account for these variables when conducting analyses on how different anxiety levels impact performance.

The fourth point is that the Virtual Reality Protocol was standardized and was not customized to suit the individual’s needs. To ensure internal consistency there was one natural video used. It remains to be determined if VR’s efficacy varies with the type of content, the duration, or interactivity. Further research is necessary on personalized or adaptive VR Experiences.

Fifth, cortisol levels were measured shortly after the intervention, which may not fully reflect the delayed response of the hypothalamus-pituitary-adrenal (HPA) axis. Although all samples were collected in the morning to minimize diurnal rhythm variations, diurnal rhythm fluctuations and biological variability may still affect the results.

Sixth, this study did not include postoperative follow-up, therefore it was impossible to assess whether the anxiolytic effect of virtual reality (VR) persisted during the recovery period. Future research should include longitudinal follow-up to assess the duration and clinical significance of the observed anxiety reduction.

Seventh, the operative duration varied between 20 and 100 min, which may have affected anticipated stress and biomarker levels. Although the surgical type was standardized (extraction of mandibular wisdom teeth under general anesthesia), operative duration was not considered in the analysis. Future research should include operative duration as a covariate.

Eighth, this intervention was only tested in patients undergoing wisdom tooth extraction under general anesthesia. While this standardized the surgical procedure, it may limit its applicability to other surgeries, anesthesia techniques, or degrees of invasiveness.

Ninth, the lack of specific scales for measuring dental anxiety (such as the MDAS or Corah’s DAS) may have affected the variability of salivary biomarkers. This suggests that future research should employ multiple tools to assess anxiety.

High Cohen’s d (and large effect) values for some of the post-intervention comparisons may indicate considerable sampling variability and variance structure as a pilot study will typically include an inadequate sample size. Thus, researchers should tentatively accept this information until validated by research using larger sample sizes.

Finally, the control environment itself—a quiet room with low lighting and minimal stimuli—may have exerted a calming effect on patients, thereby reducing the observed contrast between the groups and potentially underestimating the full impact of VR.

## Conclusion

This randomised pilot trial suggests that a 15 min VR intervention is able to reduce self-reported preoperative anxiety as demonstrated by a statistically significant Group x Time interaction for APAIS. Both salivary cortisol and alpha-amylase levels generally decreased with time; however, there was no evidence of any significant Group x Time interaction, therefore no definitive conclusion about the efficacy of VR as compared to routine care could be made at this time. Further randomised studies are needed to determine if this finding is replicable with larger sample sizes, additional data collection points and more refined control groups.

## Data Availability

The datasets generated and/or analyzed during the current study are not publicly available due to confidentiality and participant privacy constraints. However, the data are available from the corresponding author on reasonable request and with appropriate institutional approval.

## Declarations

### Clinical Trial Number

INA-28369A7.
